# Inducing gender/professional identity compatibility promotes women’s compensation requests

**DOI:** 10.1371/journal.pone.0207035

**Published:** 2018-11-12

**Authors:** Shira Mor

**Affiliations:** Faculty of Business, The Israeli Center for Business and Law, Ramat Gan, Israel; Kansas State University, UNITED STATES

## Abstract

In this paper, I examine whether inducing gender/professional identity compatibility prior to a self-advocacy negotiation, may enhance women’s assertiveness in a compensation negotiation and mitigate potential social backlash concerns for assertiveness. In two experimental lab studies where women negotiated with a male counterpart as sellers and job candidates, I found evidence supporting the causal link between state gender/profession identity integration and higher levels of assertiveness in women’s self-advocacy compensation negotiations.

## Introduction

Gender inequalities in pay are at the forefront of international debate. In 2014, the abrupt firing of Jill Abramson—the former executive editor of the New York Times—triggered a widespread debate about the negative consequences of high levels of assertiveness in self-advocacy negotiations among professional women (Covert, 2014 [1]). A few months earlier, Germany faced a large public debate when Angela Merkel introduced gender quotas in supervisory boards as a way of tackling the underrepresentation of women in senior management (Oltermann & Neate, 2013 [2]). Recent advancement in theory suggest that gender/profession identity integration- the perceived compatability between feminine-typical and professional typical behaviors- may be one factor that can explain variability in women’s inclination to display gender typical (e.g. feminine attributes) and professional typical behaviors (e.g. assertiveness). The use of assertiveness among professional women has been previously linked with fear of backlash for high levels of assertiveness in self-advocacy negotiations (see Amanatullah & Morris, 2010 [3]; Bowles, Babcock & Lai, 2007 [4]), and thus are relevant for our theorizing about the psychological effects of inducing gender/professional identity integration.

In the context of women and workplace, identity integration refers specifically to the perceived compatability between gender and professional prescriptive roles (e.g. the ability to enact both roles simultaneously). For example, to what extent does feminine-typical traits are perceived as facilitating (rather than inhibiting) of enacting professional typical traits such as high levels of assertiveness. Identity integration is an individual difference measure which assesses the degree to which dual identities are experienced as compatible (e.g. facilitating one another) as opposed to conflicting (Benet-Martinez, & Haritatos, 2005 [5]; Benet-Martinez, Leu, Lee & Morris, 2002 [6]; Cheng, Sanchez-Burks & Lee, 2008 [7]; Mok & Morris, 2012a; 2012b [8,9]; Sacharin, Lee & Gonzalez, 2009 [10]; Wallen, Mor, & Devine, 2014 [11]). The approach of measuring trait identity integration relies on a self-report measure which consists of conflict and compatability subscales. Past research revealed that trait level identity integration is positively associated with female engineers’ creative performance; female engineers with higher gender/profession identity (GPII) designed more creative products for female consumers (Cheng et al., 2008 [7]). Other studies examining gender/profession identity integration among business women reveals that when primed with a professional context, women with higher identity integration adopt an orientation that is consistent with the demands of a professional task whereas women with less integrated identities adopt an orientation that contrasts with the professional task demands. More recent research examining female lawyers with more integrated gender/professional identities reveal that women with higher gender/profession identity integration assimilate their behavior to the demands of the professional contexts (Mok & Morris, 2012a [8]). If female engineers who are higher on identity integration assimilate more to the demands of the professional task, the question remains if women high on identity integration may also use greater assertiveness when negotiation economic compensation on their own behalf? This is the research question the present research aims to test. Since professional typical behaviors in the context of competitive bargaining are associated with masculine-typical behaviors such as high assertiveness (associated with backlash for professional women), we would expect that women higher on identity integration will feel more uninhibited to display both feminine-types and professional-typed negotiation (e.g. assertive) behaviors, an thus would be more likely to demand for higher economic outcomes when negotiating their own compensation.

Another related question is whether identity integration reduces women’s anticipated backlash, a factor identified to reduce women’s assertiveness in self-advocacy in negotiations (Amanatullah & Morris, 2010 [3]). More nascent research reveals that this may be plausible as social backlash is absent among women higher on gender/profession identity integration despite their use of dominance in competitive negotiations (Mor, Mehta, Fridman, & Morris, 2014 [12]). Thus, we further suggest that inducing gender/profession identity compatibility among women may also alleviate anticipated social backlash for women’s compensation requests in negotiations. To test my hypotheses, I induced gender/professional identity integration among business women in a laboratory setting prior to making first offers and asking in a self-advocacy buyer-seller and salary negotiation.

### Inducing identity compatibility

To test my hypotheses, I manipulated gender/profession identity integration using a recall task. Recent studies reveal that identity integration is not only a stable individual difference, but also a psychological state that can shift when recalling past experiences associated with both social identities (Cheng & Lee, 2009 [13]; Cheng & Lee, 2013 [14]). Adapting this paradigm to a negotiation setting, we expected that inducing high identity integration in women prior to a competitive price and salary negotiation would increase women’s asking. I did so by assessing women’s first offers as sellers. To determine the desired sample size to detect a medium size effect observed (b = .49, desired sample size with 95% power: 41 required participants to achieve MDE) in previous gender/profession identity integration research (Mor et al., 2014 [12]; Wallen et al., 2014 [11]), I used G power—a recommended statistical software for behavioral scientists (Faul, Erdfelder, Lang, & Buchner, 2007 [15]).

## Materials and methods

### Study 1: Price negotiation

#### Method

**Participants:** One hundred and ten women majoring in business were recruited from a Dutch business school for a lab study. Participants in the laboratory signed informed consent forms in verbal form. Erasmus University’s Review Board approved the materials and procedures prior to the study. The materials were presented in English to increase the number of eligible participants. Seven participants did not understand the case materials instructions in English and were removed from the final dataset. The final dataset included 103 women. Participants represented women from 33 nationalities (*M*_age_ = 21, 41.8% working part-time/full-time). Participants self-reported the following nationalities: 52.4% Dutch, 12% Chinese, 10% German, and 24.6% represented 1–2 women from diverse European nationalities as well as one South Korea participant. The descriptive statistics are reported in Table 1.

**Table 1:** Descriptive statistics for key variables in Study 1.

Study 1. Descriptive Statistics and Correlations Amongst Target Level Variables											
		Control Condition (n = 50)					Integration Condition (n = 53)				
		*M*	*SD*	Median	5th Percentile	95th Percentile	*M*	*SD*	Median	5th Percentile	95th Percentile
	**Key Predictors**										
1	Age	20.98	2.03	20.50	18.00	25.00	20.77	1.85	21.00	18.00	24.00
2	Employed (Yes = 1; No = 0)	0.36	0.48	0.00	0.00	1.00	0.47	0.50	0.00	0.00	1.00
3	First Offer	23.56	7.38	25.00	7.75	36.35	26.67	5.67	26.00	18.70	35.90
4	Anticipated Backlash	21.32	6.27	21.50	6.65	32.90	23.18	5.70	21.00	18.70	36.50

**Procedure:** Participants were invited to participate in a lab study for women in business.

**Manipulation:** Participants were then randomly assigned to either an identity compatibility or experimental condition. The manipulation in the identity compatibility condition instructed women to reflect on their personal experiences and describe a professional situation in which gender and professional identities/roles complemented (e.g. facilitated rather debilitated) each other. The instructions were as follows:

“In the next section we would like you to take a few moments to reflect about the positive aspects of being a female professional. Please list three experiences/situations in which you felt your female role facilitated/complemented your professional role”

Participants in the control condition were asked to recall an experience of managing their time surfing the internet and completing coursework using the following prompt:

“In the next section we would like you to take a few moments to reflect how you manage your time between surfing the internet and completing course related work. Please list three experiences/situations describing how you manage your time.”

**Negotiation Task:** Next, participants were advised they are going to do a short negotiation. They were advised that their negotiation counterpart (the buyer) is called Robert and were presented with a picture of a mid-aged business man in a suit. Participants then read their role (the seller) and were asked to read the case background materials (Synertech Dosagen, see Galinsky & Mussweiler, 2001 [16]). The negotiation involved the purchase of a pharmaceutical plant in millions (dollars). Participants were advised that both parties (e.g. the buyer and seller) were given the same general information. Below is a summary of the general information provided to participants:

“Two years ago, the Dosagen plant was appraised at $19 million. The local real estate market has declined 5% since then. However, the Dosagen plant is a unique property and general real estate trends may not apply. A plant similar to the Dosagen plant, although newer, sold for $26 million nine months ago…If you shut down the plant, strip it, and then sell the plant and equipment separately, you estimate you would net a minimum of $17 million and that it would take you three months to do so. Your preference is to sell the plant in its current configuration, and to a buyer who would keep the current high quality, biotech experienced work force… you are about to meet Robert Graham, the CFO of Synertech. You have full authority to sell the plant for whatever you can get. The price must be in cash. No other terms can be added to the negotiation.”

**Negotiation Goals:** Participants were first asked to report their aspiration goals (e.g. reservation point and aspiration goal) for the upcoming negotiations in dollar amounts using an open-ended text format. The same format also applied to the items below.

**First Offer:** Participants were then asked to indicate what would be their first offer to the buyer (Robert): “if you are given the opportunity to make the first offer in this negotiation, how much will you suggest Robert to pay?”

**Anticipated Backlash:** Next, participants were asked to report how much they think they can ask for from Robert without incurring social backlash (see Amanatullah & Morris, 2010 [3]): “How much do you think you can reasonably ask for without Robert thinking you are a pushy person?” and “How much do you think you can reasonably ask for without causing Robert to punish you for being too demanding?”. These two measures were highly correlated *(r* = .81, *p* = .000) and therefore were averaged to create our measure of anticipated backlash (this procedure is consistent with the measure reported by Amanatullah & Morris, 2010 [3]). At the end, participants then answered a number of questions about their intended negotiation behaviors.

**Manipulation check and Demographics:** Participants were then asked a number of questions about their anticipated behavioral goals in anticipation for the negotiation. At the final part, participants were asked to recall the recall task they had completed earlier and asked to report a number of demographic questions. At the end of the study participants were thanked and paid.

### Results

#### First offers

In line with my assumptions, women in the identity/integration condition made higher first offers as sellers (*M* = 26.68) compared to women in the control condition (*M* = 23.56), F (1, 101) = 5.81, *p* = .02, η^2^ = .05. I further examined whether women’s first offers in this case was parallel to men’s first offers in this case by using first offer estimates from a recently published study (Gunia, Swaab, Sivanathan, & Galinsky, 2013[17]). The results revealed that women’s first offers in the control condition were significantly lower (*M* = 23.56) than men’s reported first offers (*M* = 26.20), t (49) = -.25, *p* = .02. At the same time, women’s first offers in the identity integration condition (*M* = 26.68) did not differ significantly from men’s first offers (*M* = 26.20), t (52) = .62, *p =* .*54*. These results suggest that priming identity integration among women may reduce the gender pay gap as higher first offers by sellers lead to a higher final settlement price (Galinsky & Mussweiler, 2001 [16]).

#### Anticipated backlash

Next, I examined anticipated backlash for self-advocacy as sellers. Women in the identity integration condition revealed a trend where they reported a higher price as sellers without incurring social backlash (*M* = 23.18) relative to women in the control condition (*M* = 21.32), F (1,101) = 2.51, *p* = .11, η^2^ = .02. The effect of identity integration condition on the backlash threshold did not meet statistical significance. In the next study, I increased the sample size to increase statistical power and also tested the hypotheses in a salary negotiation which carried more heavy social costs for women’s asking—women’s salary negotiations.

### Study 2: Salary negotiation

#### Method

**Participant:** One hundred and thirty-five female participants from a Dutch business school were recruited for a lab study. The materials were presented in English as in Study 1. Seven participants were removed from the final dataset as they failed to comprehend the case materials and instructions in English. The final dataset was composed of one hundred and twenty eight women from business majors representing 30 nationalities (*M*_age_ = 21, 53.9% working part-time/full-time). Participants represented the following self-reported nationalities: 45.3% Dutch, 13.3% Chinese, 9.4% German, 32% represented 1–2 women from diverse European nationalities as well as one South Korean and one Taiwanese). The descriptive statistics are reported in Table 2.

**Table 2:** Descriptive statistics for key variables in Study 2.

		Control Condition (n = 65)					Integration Condition (n = 63)				
		*M*	*SD*	Median	5th Per.	95th Per.	*M*	*SD*	Median	5th Per.	95th Per.
	**Variables**										
1	Age	21.14	2.50	20.00	19.00	25.00	21.67	2.61	21.00	19.00	26.00
2	Employed (Yes = 1; No = 0)	0.49	0.50	0.00	0.00	1.00	0.58	0.49	1.00	0.00	1.00
5	First Offer	37476	3648	37000	33150	43700	38017	4176	38000	35000	45000
6	Anticipated Backlash	34045	3591	34450	31300	40000	35679	3769	35000	32000	43700

**Procedure:** Participants were invited to participate in lab study for women in business.

**Manipulation:** Participants were then randomly assigned to either an identity compatibility or control condition. The manipulation in the identity compatibility condition instructed women to reflect on their personal experiences and describe a professional situation in which gender and professional identities complemented each other. The instructions were as follows:

In our past studies with female business professionals, many participants have told us it is easy to manage the two identities-- female and business professional. Some ways they have described the two identities include the following:

- "My female side is compatible with my business professional side"
- "I feel that my female and business professional sides are complementary"

Pick a description above that most closely relates with your experience; place a check-mark next to that description. Then, in space below, describe how that description relates with your experience in a professional business context. Give an example.

Participants in the control condition were asked to recall an experience of managing their different vacation times during the year using the following prompt: In our past studies, participants reported how they manage their vacation time during Christmas and summer. Some ways they have described it include the following:

- "I try to take time off in Christmas and summer"
- "I try to find new destinations for travel in Christmas and summer"

Pick a description above that most closely relates with your experience; place a check-mark next to that description. Then, in space below, describe how that description relates with your experience. Give an example.

**Negotiation Task:** Next, participants were advised their negotiation counterpart (the recruiter) is called Daan Smitt and were presented with a picture of a mid-aged business man in a suit. Participants then read their role (job candidate for a consulting firm) and were asked to read the case background materials. Below is a short summary of the information provided to job candidates about the salary negotiation task the salary ranges in their industry:

“In preparation for today’s negotiation you have done a lot of research, including googling average starting salaries in this industry. You have learned that new hires in consulting that have education and experience similar to yours, average around **€33,043** a year. Some additional statistics you were able to find were from analysts in the industry reporting their starting salaries. You found that the range of salaries for a starting analyst was between €21,000 (e.g. lowest) and €42,000 (e.g. highest) a year.”

**Negotiation Goals:** Participants were first asked to set their goals for the negotiation in euros using the same items as in Study 1.

**First Offer:** Participants were asked to indicate the euro amount of first offers using the following statement: **“**If you are given the opportunity to make the first offer in this negotiation, how much will you suggest Daan pay you as your starting salary?”

**Anticipated Backlash:** Next, participants were asked to report the euro amount they think they can ask for from the recruiter (Daan) without incurring social backlash using the following items: “How much do you think you can reasonably ask for without Daan thinking you are a pushy person?” and “How much do you think you can reasonably ask for without causing Daan to punish you for being too demanding?”. As in Study 1, these two items were averaged to create our measure of anticipated backlash (*r* = .73, *p* = .00).

**Manipulation check and Demographics:** Participants were then asked a number of questions about their anticipated behavioral goals and emotions in anticipation for the negotiation. At the final part, participants were asked to recall the recall task they had completed earlier and asked to report a number of demographic questions. At the end of the study participants were thanked and paid.

#### Results

**First Offer:** Women in the gender/professional identity compatibility condition made the recruiter a higher first offer (*M* = € 38,016.56) than the average market price reported in the materials (€33,043), however, their first offers did not differ significantly from offers of women in the control condition (*M* = €37,476.15), F (1,126) = .61, *p* = .44, η^2^ = .00.

**Anticipated Backlash:** In line with our predictions, women induced to think about gender/profession compatibility prior to their salary negotiation reported they could ask the recruiter for a higher salary (e.g. a 5% increase in salary) without incurring social backlash (*M* = €35,769) compared to women in the control condition (*M* = €34,045), F (1,126) = 7.02, *p* = .01, η^2^ = .05. These results supported our hypotheses that identity integration reduces anticipated backlash when women self-advocate for their own salaries.

## Discussion

The present studies provide an empirical contributions to ongoing research as they unlock one factor that can unleash women’s compensation requests. The results from the two experimental studies provide coherent and consistent empirical evidence supporting a causal association between gender/profession identity integration and women’s compensation negotiations. Moreover, gender/profession identity integration was causally associated with reduced anticipated social backlash, but only during women’s self-advocacy salary negotiations. At the same time, while identity integration reduced fear of social backlash in self-advocacy negotiations, this reduced fear did not directly translate into women’s compensation requests. These results extend recent findings revealing that women high in trait level gender/profession identity integration are less likely to incur social backlash in self-advocacy negotiations (Mor et al., 2014 [12]). The present findings also extend prior work revealing that women experience heightened anticipated backlash in self-advocacy roles (see Amanatullah & Morris, 2010 [3]) by suggesting that these concerns could be diminished if women perceive their gender and professional roles to be highly compatible. At the same time, due to the fact that reduced social backlash concerns did not consistently affect women’s compensation requests, future research and theorizing is needed to further investigate the boundary conditions for the relationship between anticipated social backlash concerns and women’s assertiveness in self-advocacy compensation negotiations.

Despite the advantages of the present approach it suffers from some limitations. First, the hypothetical experiments had no stakes and did not take place face-to-face negotiations, and furthermore, we did not include any explicit questions on feelings of identity integration as manipulation checks to prevent any demand characteristics. However, these limitations may suggest that the effects observed in this weak situation (e.g. where demand characteristics were small), may be even more pronounced in real-world negotiations. Moreover, future research may do well by adapting implicit manipulation checks for the identity integration primes, to circumvent any demand characteristics by participants to report high levels of identity integration. Second, we did not include male participants in our identity integration studies, however, theoretically, we would expect that this construct would be most relevant for men in female dominated fields (see Wallen at al., 2014 [11]) rather than business. Third, I did not explore whether there were any racial or ethnic differences in the performance of White versus minority women as this can only be done with a larger sample. Emerging evidence reveals that gender-based social backlash effects documented with White targets are reversed when the targets are Black (Livingston, Rosette, & Washington, 2012 [18]) or absent among Asian women (Toosi, Mor, Semnani-Azad, Phillips, Amanatullah, 2018 [19]). Thus, I suggest that future research should examine race and gender intersectionality. Another potential avenue for future research is examining whether inducing identity integration can affect women’s compensation requests in professions where gender parities are most extreme, such as finance or consulting (see Barbulescu & Bidwell, 2013 [20]; Bowles et al., 2005[21]). In summary, the present research provides new clues to why some professional women may be more inclined to display assertiveness in self-advocacy negotiations in the workplace and have reduced concerns about social backlash- the psychological state of perceiving greater overlap between gender and professional roles.
